# Effects of anti‐human T lymphocyte immune globulins in patients: new or old

**DOI:** 10.1111/jcmm.12860

**Published:** 2016-04-15

**Authors:** Diane C. Wang, Xiangdong Wang, Chengshui Chen

**Affiliations:** ^1^ Department of Respiratory MedicineThe First Hospital of Wenzhou Medical UniversityWenzhouChina

**Keywords:** transplantation, anti‐human T lymphocyte immune globulins, patients, immune, therapy

## Abstract

Multiple studies demonstrated that anti‐human T lymphocyte immune globulins (ATG) can decrease the incidence of acute and chronic graft rejection in cell or organ transplants. However, further in‐depth study indicates that different subgroups may benefit from either different regimes or alteration of them. Studies among renal transplant patients indicate that low immunological risk patients may not gain the same amount of benefit and thus tilt the risk *versus* benefit consideration. This may hold true for low immunological risk patients receiving other organ transplants and would be worth further investigation. The recovery time of T cells and natural killer (NK) cells also bears consideration and the impact that it has on the severity and incidence of opportunistic infections closely correlated with the dosage of ATG. The use of lower doses of ATG in combination with other induction medications may offer a solution. The finding that ATG may lose efficacy in cases of multiple transplants or re‐transplants in the case of heart transplants may hold true for other transplantations. This may lead to reconsideration of which induction therapies would be most beneficial in the clinical setting. These studies on ATG done on different patient groups will naturally not be applicable to all, but the evidence accrued from them as a whole may offer us new and different perspectives on how to approach and potentially solve the clinical question of how to best reduce the mortality associated with chronic host‐*versus*‐graft disease.

## Introduction

Graft‐*versus*‐host disease (GVHD) occurs in both acute and chronic forms and poses as a major complication following hemopoietin cell transplantation (HCT) and organ transplantation, which leads to an increase in mortality and decreased quality of life. 1 Acute GVHD with donated stem cells with high quantities of T cells of peripheral blood as the source is considered as one of the risk factors of chronic GVHD. The anti‐human T lymphocyte immune globulins (ATGs) as ‘T‐cell‐depleting’ antibodies were suggested to decrease the incidence of GVHD. Anti‐human T lymphocyte immune globulins is derived from multi‐sources, of which thymoglobulin is a polyclonal ATG (rATG) from immunizing pathogen‐free rabbits with fresh human thymocytes. Most studies are performed using rATG, since ATGs derived from different animals share only some common features and have yet to be compared in a controlled environment. Anti‐human T lymphocyte immune globulin–based therapies have been incorporated into immunosuppressive regimes for years, but debate is still ongoing regarding appropriate time of treatment, therapeutic window and dosage. Appropriate dosing and timing would enable the usage of the lowest dose possible while still achieving optimal clinical efficiency and minimizing possible side effects, such as opportunistic infections, loss of transplant‐*versus*‐leukaemia effect and *de novo* post‐transplant cancers.

The regulation of the proliferation and function of lymphocytes and peripheral blood mononuclear cells has been suggested as an important mechanism by which ATG treats the rejection of organ transplantation 2. Anti‐human T lymphocyte immune globulins have multi‐effects to directly or indirectly influence in the interaction between cells through the regulation of cytokine and chemokine production from lymphocytes. The present commentary recalls the special attention from clinicians and clinical scientists to results from recent clinical trials on therapeutic effects of ATG and headlights new understanding of therapeutic strategy. New findings shall lead scientists to new questions and mechanisms by which ATG targets and changes molecular and cellular signals within cells and tissues.

### Effects in stem cell transplantation

Therapeutic effects of ATG in chronic GVHD after HCT are confirmed by a number of clinical trials, while suggested risk factors or mechanisms should be reconsidered. Kroger *et al*. conducted a multicentre, prospective phase 3 study to investigate whether the inclusion of rATG in a myeloablative conditioning regimen, including cyclophosphamide, total body irradiation or busulfan, with or without etoposide, would decrease the risk of chronic GVHD in patients who were in complete remission from acute leukaemia following a peripheral blood stem cell transplant from a human leucocyte antigen‐identical sibling 3. Results after 2‐year follow‐up noted no significant differences in rates of relapse, infectious complications, acute GVHD or other negative events in the completed trial with 155 cases. Of specific note is that no significant difference in rate of relapse, potentially caused by loss of graft‐*versus*‐leukaemia effect following T‐cell depletion. However, the cumulative incidence of chronic GVHD was significantly improved to 32% in patients with acute GVHD treated with ATG, as compared with 69% without ATG. It indicates that ATG can increase 2‐year survival from chronic GVHD, which is one of the main causes of increased mortality after HCT 4. More than 90% of patients with ATG were able to stop using cyclosporine within 1 year compared with 39% without ATG. Prior to this, the main studies showing a beneficial effect of ATG in HCT from human leucocyte antigen‐identical siblings have been smaller, retrospective studies.

The effect of rATG inclusion in the induction therapy (*e.g*. either cyclosporine or tacrolimus with either methotrexate or mycophenolate) for patients with HCT could increase the number of patients to stop immunosuppressive treatment within 1 year. A follow‐up study showed that 52.9% of patients with ATG, in contrast to 16.9% without ATG, could remain immunosuppressive drugs free for 3 years 5. The incidence of acute GVHD was lower in the group receiving ATG with an earlier onset but the severity was similar. Anti‐human T lymphocyte immune globulins therapy could clearly reduce the incidence and severity of chronic GVHD within 12 months, but not the incidence of serious adverse effects. Seven different questionnaires were used to evaluate patient‐reported outcome of quality of life in patients who survived without relapse to 12 months 6. The results from the Lee Scale indicated a significantly reduced symptom burden from chronic GVHD, as did the Atkinson Life Happiness Scale. The other five questionnaires remained equivalent but did not report any adverse effects on the symptom burden at 12 months 6.

### Effects in kidney transplantation

Anti‐human T lymphocyte immune globulin remains the most commonly used induction treatment for renal transplants with over 60% in the United States alone being used for *de novo* kidney transplants 7. The level of T‐cell depletion and incidence of side effects are highly dose dependent, while the efficacy of high and low doses showed comparable. One study in 1998 showed a similar outcome profile in high‐risk immunological patients given an average of 5.7 mg/kg with those given 10.3 mg/kg 8, while another study on low‐risk patients in 2014 showed low biopsy‐proven acute‐rejection rates with a lower incidence of opportunistic infections in the lower 2.25 mg/kg group when compared to patients who had received 3.75 mg/kg 9. Such difference between studies can be explained by many factors, while should be considered to be further validated by the precision of advanced measurements and molecular pharmacology and toxicology 10, 11, 12, 13, 14 or the application of well‐identified biomarkers 15, 16, 17, 18. Kho *et al*. studied the effect of different rATG doses and found that T cells and NK cells remained depleted at 1‐week post‐transplant after having been given 1.5 mg/kg prior to organ transplant, but recovered to baseline over the course of 1 month. However, patients who received 3 mg/kg were noted to have continued depletion at 1 month and recovered to baseline at 1 year. Patients receiving 6 mg/kg had T‐cell and NK cell depletion for over 1 year 19.

Three hundred nine patients were studied following *de novo* low‐risk immunological kidney transplants from deceased donors across multiple centres in a randomized trial 20. The results of this study showed a decrease in biopsy‐proven acute rejections when having received an induction therapy of rATG (1.25 mg/kg) followed by a tacrolimus triple–based therapy consisting of calcineurin inhibitors (CNI), an anti‐proliferative and steroids, instead of solely tacrolimus triple–based therapy, but at 1 year the renal function, patients‐and‐graft‐survival rates and delayed graft function were similar between the groups. However, an increased incidence of side effects, such as leucopenia, thrombocytopenia and cytomegalovirus infections, has been noted in both groups indicating that low‐risk patients receiving tacrolimus‐based triple therapy may not need rATG induction. These results raise the question of which patients groups and risk factors would benefit from rATG and at which doses.

### Effects in heart transplantation

Induction therapy prior to heart transplantation has also had significant implications to reduce the rates of rejection and enable delaying the initiation of CNIs. Current treatment protocols consist of a polyclonal anti‐lymphocyte antibody, such as rATG, or an anti‐interleukin 2 monoclonal antibody, such as basiliximab. Studies have indicated that basiliximab is well tolerated as an induction therapy for heart transplantation with a preferable side‐effect profile 21, while ATG could shield better against acute rejection following heart transplantation 22. Ansari *et al*., using the International Society for Heart and Lung Transplantation database, studied data from 42,474 cases of adult heart transplantation to compare the short‐term and long‐term effects of basiliximab *versus* ATG 23. Their results demonstrated that effects of basiliximab for induction were associated with poorer long‐term survival rates at 5 and 10 years when compared to the cohort who had used ATG in the induction regime. This association was due to a higher risk of cardiovascular events, graft failure and infection in the basiliximab group. However, the ATG cohort did show a higher incidence of malignancy‐related deaths after 7 years. Those findings held true for most of the subgroups apart from the group who had had other transplants or re‐transplants. In this latter group, basiliximab had a similar performance profile as ATG. This may be the effect of sensitization of previous ATG treatment resulting in ATG antibodies, which in turn may result in decreased active thymoglobulin levels 24.

### Potential toxic effects

The severity and incidence of cytomegalovirus infections have been linked to the dosage of rATG, *e.g*. lower doses having lower occurrence and less severity. Ganciclovir has been used as a prophylactic treatment in concordance with rATG, but a study has indicated that rATG induction, together with low‐dose tacrolimus and everolimus, was still clinically efficient while having a low incidence of cytomegalovirus disease even without the prophylactic treatment *versus* induction with basiliximab‐, tacrolimus‐ and mycophenolate mofetil‐based immunosuppression (10% *versus* 37%) 25.

The use of rATG may lower the dose needed of the CNIs which themselves carry a side‐effect profile and risk of nephrotoxicity and the weighing of the risks and gains will vary depending on the most dangerous or most likely side effects for patients. Complete withdrawal of CNIs is still under investigation. Some evidence indicate that such strategy would be safe in case of risk factors for delayed graft function, *e.g*. older donor age, long ischaemia time or a vascular donor 26. Another way of altering the regimen would be the duration of the different medications in the combination. Some evidence from a few prospective, randomized trials indicates that maintenance immunosuppressive therapy with CNIs, mycophenolate and rATG induction would allow the withdrawal of steroids within the first 3 months 27. This would in turn reduce the likelihood of side effects from long‐term steroids, such as hypertension, weight gain and diabetes. The likelihood of *de novo* cancers following rATG treatment has remained a concerning possibility. American and European registered study showed that rATG was not associated with lymphoma or any other solid cancer apart from melanoma in the non‐Hispanic white population 28. However, this may be a result of the fact that the rATG doses have been decreasing in order to minimize the dose‐dependent side effects. There is also an urgent need to develop drug‐specific biomarkers to monitor and reflect drug efficacy, toxicity and metabolism through the application of gene or protein screening. Disease biomarkers, network biomarkers, and dynamic network biomarkers of drug‐targeted driver genes or factors can be identified and validated by gene/protein expression, gene sequencing, epigenetics and biological functioning 29, 30, 31, 32.

## Conclusion

Multiple studies have shown that ATG can decrease the incidence of both acute and chronic graft rejection in blood stem cell, heart and renal transplants. However, further in‐depth study indicates that different subgroups within these cohorts may benefit from either different regimes or alteration of them. Studies among renal transplant patients indicate that low immunological risk patients may not gain the same amount of benefit and thus tilting the risk *versus* benefit consideration. This may hold true for low immunological risk patients receiving other organ transplants and would be worth further investigation. The recovery time of T cells and NK cells also bears consideration and the impact that it has on the severity and incidence of opportunistic infections. This is also closely correlated with the dosage of ATG used. The use of lower doses of ATG in combination with other induction medications may offer a solution to this. The finding that ATG may lose efficacy in cases of multiple transplants or re‐transplants in the case of heart transplants may very well hold true for other transplantations as well. This may lead to reconsideration of which induction therapies would be most beneficial in the clinical setting. These studies on ATG done on different patient groups will naturally not be applicable to all, but the evidence accrued from them as a whole may offer us new and different perspectives on how to approach and potentially solve the clinical question of how to best reduce the mortality associated with chronic host‐*versus*‐graft disease.

## Conflict of interest

The authors declare no competing of interests.
